# Supplementary material for: Dynalign II: common secondary structure prediction for RNA homologs with domain insertions
Source: Nucleic Acids Res. 2014 Nov 21;42(22):13939–48. doi: 10.1093/nar/gku1172 (PMC4267632; doi:10.1093/nar/gku1172)
Supplement: SUPPLEMENTARY DATA [file supp_gku1172_nar-02021-z-2014-File012.zip › manual/RNA_class/html/thermodynamics_8h.html]

RNAstructure Classes: F:/RNAstructure-co-from-repository/RNAstructure/RNA\_class/thermodynamics.h File Reference

- Main Page
- Classes
- Files

- File List
- File Members

# F:/RNAstructure-co-from-repository/RNAstructure/RNA\_class/thermodynamics.h File Reference

`#include "../src/rna_library.h"`  

|  |  |
| --- | --- |
|  |  |
| Classes | |
| class | Thermodynamics |
|  | Thermodynamics Class. More... |
| Defines | |
| #define | THERMODYNAMICS\_H |
| #define | TOLERANCE   0.01 |

---

## Define Documentation

#define THERMODYNAMICS\_H

#define TOLERANCE   0.01

---

Generated on Tue Jul 30 10:52:29 2013 for RNAstructure Classes by 
 1.5.7.1
